# Dissociation in How Core Autism Features Relate to Interoceptive Dimensions: Evidence from Cardiac Awareness in Children

**DOI:** 10.1007/s10803-019-04279-4

**Published:** 2019-11-09

**Authors:** E. R. Palser, A. Fotopoulou, E. Pellicano, J. M. Kilner

**Affiliations:** 1 Department of Clinical, Educational and Health Psychology, Psychology and Language Sciences, University College London, Gower Street, London, WC1E 6BT UK; 2 Department of Clinical and Movement Neurosciences, UCL Institute of Neurology, University College London, 33 Queen Square, London, WC1N 3BG UK; 3 Centre for Research into Autism and Education, UCL Institute of Education, University College London, London, UK; 4 Department of Educational Studies, Macquarie University, Building X5B, 29 Wally’s Walk, Room 209, Sydney, NSW 2109 Australia; 5 Present Address: Sandler Neurosciences Center, Department of Neurology, University of California San Francisco, 675 Nelson Rising Lane, Suite 190, San Francisco, CA 94158 USA

**Keywords:** Autism, Interoception, Social Cognition, Affect

## Abstract

Interoception in autism is receiving increasing research attention. Previously, differences were identified in autism on both objective and subjective measures of interoception, and an association with anxiety. Yet, it is currently unknown how interoception relates to core autism features. Here, in 49 autistic children, we consider how interoceptive accuracy (measured with heartbeat detection tasks) and sensibility (subjective judgements of awareness) relate to overall severity on the Autism Diagnostic Observation Schedule, and symptom domains of social-affective and repetitive, restricted behaviors. Socio-affective features were related to interoceptive sensibility, while repetitive restricted behaviors were related to interoceptive accuracy. This dissociation suggests disparate interoceptive mechanisms for the formation and/or maintenance of autistic features.

**Electronic supplementary material:**

The online version of this article (10.1007/s10803-019-04279-4) contains supplementary material, which is available to authorized users.

## Introduction

Interoception describes the perception and integration of autonomic, hormonal, and homeostatic signals that collectively represent the physiological state of the body (Barrett and Simmons 2015), through the continuous dynamic feedback of afferent visceral signals to the brain (Critchley and Harrison 2013).The personal accounts of autistic individuals and their caregivers suggest a range of pervasive difficulties with the awareness and integration of interoceptive signals (Elwin et al. 2012; Gerland 1997; Musser 2018). Many of these accounts document a lack of awareness of hunger, thirst, pain and the need to make bowel or bladder movements. Difficulties with distinguishing between emotional and interoceptive states are also described (Musser 2018). Within the last few years, direct empirical investigations of interoceptive processing have emerged, which generally support the idea that interoception is atypical in autism (see DuBois et al. 2016, for review, although see Nicholson et al. 2018, Schauder et al. 2015, and Shah et al. 2016, for reports of intact interoception).

Recently, Garfinkel and colleagues proposed a three-dimensional construct of interoception (Garfinkel and Critchley 2013; Garfinkel et al. 2015). Within this model, paradigms that objectively quantify participants’ performance in detecting internal events represent measures of ‘interoceptive accuracy’ (Garfinkel et al. 2015). Questionnaire based self-report measures, and confidence judgements on objective tasks, are thought to quantify an individual’s subjective awareness of interoceptive signals and are termed ‘interoceptive sensibility’. Finally, the trial-wise correspondence between performance and confidence on objective tasks, referred to as ‘interoceptive awareness’, is thought to reflect an individual’s metacognitive insight into their interoceptive ability. This framework outlined how individuals could diverge on subjective and objective measures of interoceptive functioning.

To date, only a few studies have simultaneously considered multiple interoceptive dimensions in autism (Garfinkel et al. 2016; Mul et al. 2018; Palser et al. 2018a). In the main, these studies suggest reduced objective interoceptive accuracy with concomitant heightened interoceptive sensibility in autistic adults *and* children. Of note, when more mindfulness-related measures of interoceptive sensibility that focus on the effective interpretation of interoceptive signals are employed, such as the Multidimensional Assessment of Interoceptive Awareness (MAIA, Mehling et al. 2012), reduced interoceptive sensibility has been reported (Mul et al. 2018). As such, interoceptive sensibility in autism likely varies according to the measure employed. Presently, these questionnaires are all considered to represent the construct of interoceptive sensibility, but in the future it is conceivable that a dissociation will be argued for.

Garfinkel and colleagues further conceptualized the difference between interoceptive accuracy and sensibility as an interoceptive trait prediction error (ITPE), which provides a measure of whether an individual tends to overestimate or underestimate their interoceptive abilities (Garfinkel et al. 2016). Autistic adults and children have been found to differ significantly from non-autistic participants on this measure, indicating a tendency towards overestimation of their interoceptive abilities (Garfinkel et al. 2016; Palser et al. 2018a). Moreover, this measure predicts the severity of anxiety symptoms, with the most severe anxiety seen in those who most overestimate their interoceptive abilities. These findings suggest a divergence in how interoceptive dimensions relate to the co-occurring features of autism, emphasising the potential importance of considering interoceptive functioning on multiple measures.

The finding of interoceptive differences in autistic people does not always replicate (Nicholson et al. 2018; Schauder et al. 2015; Shah et al. 2016). Some researchers have suggested a divergent developmental trend in autism (Mash et al. 2017; Nicholson et al. 2019), such that differences present early in development may abate by adulthood (Nicholson et al. 2019) or that the effect of age on interoception may be moderated by cognitive ability, with a reduction in interoceptive accuracy with age in autistic children with an IQ under 115 (Mash et al. 2017). However, as yet, no longitudinal studies have been conducted on interoception in autism and it is possible that cohort effects may be driving cross-sectional observations.

Analysis protocols also likely differ between testing sites. For example, some researchers might exclude participants who cannot detect any heartbeats (e.g., Schauder et al. 2015), thereby discarding the participants with the poorest awareness of their interoceptive signals. A further possible explanation for the mixed results to date is differences in sampling characteristics between study populations—including the severity of autism-related symptoms. It is possible that those with more severe autistic features also show more severe interoceptive difficulties. Preliminary support for this possibility comes from work in neurotypical adults, which show an association between interoceptive processing and some of the core areas of cognition affected in autism, for example, a positive correlation between interoceptive accuracy and emotional theory of mind abilities (Shah et al. 2017).

Only three studies to date have adopted such an individual differences approach, with inconclusive results (Mul et al. 2018; Palser et al. 2018a; Shah et al. 2016). One study found no relationship between interoceptive accuracy and autistic traits as measured with the Autism Quotient (AQ; Baron-Cohen et al. 2001) in both non-autistic and autistic adults (Shah et al. 2016). A second found that the Awareness dimension of the Multidimensional Assessment of Interoceptive Awareness (MAIA) (comprising trusting, noticing and emotional awareness) related negatively to autistic traits on the AQ (Mul et al. 2018). Yet, the AQ has been criticized for being a poor predictor of clinically-assessed signs of autism (Ashwood et al. 2016) and therefore might not be the best measure for such an analysis. In a third study, we reported a weak negative relationship between standardized severity scores on the gold-standard Autism Diagnostic Observation Schedule (ADOS-2; Lord et al. 2012) and interoceptive sensibility and a stronger negative relationship with ITPE in a sample of 30 autistic children and adolescents (Palser et al. 2018a). No relationship, however, was found between autism severity and interoceptive accuracy.

It is also possible that interoceptive processing might vary in how it relates to the aspects of autism, such as emotion. Indeed, it has been argued that the higher rates of interoceptive differences observed in autism actually reflect higher rates of emotional difficulties in the condition, termed ‘alexithymia’, rather than autism per se (Gaigg et al. 2018; Shah et al. 2016). The salient features of alexithymia are difficulties in identifying and describing feelings, in distinguishing between feelings and bodily sensations of emotional arousal, in imagination, and an externally-oriented cognitive style (Nemiah et al. 1976). Alexithymia in autism has been found to be associated with reduced interoceptive accuracy (Shah et al. 2016), although recent null findings call this link into question (Mul et al. 2018; Nicholson et al. 2018).

The relationship between interoceptive sensibility and alexithymia appears to vary according to the measure employed. When more mindfulness-related measures of interoceptive sensibility are used, with a focus on the effective interpretation of interoceptive signals, interoceptive sensibility has been shown to be negatively correlated with alexithymia (Mul et al. 2018). Other measures of interoceptive sensibility that tap a more vigilant, anxiety-associated style have a positive relationship with alexithymia (Longarzo et al. 2015; Palser et al. 2018b), although this has yet to be replicated in an autistic population. It is possible that interoceptive differences vary according to the extent of autistic individuals’ socio-emotional difficulties. It may therefore be important to consider how interoceptive processing relates not only to overall autism severity, but also to the severity of specific features, particularly social and affective domains.

In sum, previous studies suggest that altered interoception is more likely, but not always, seen in autism than the general population, and is related to some of the co-occurring features of the disorder, namely anxiety. Yet, it is still unknown how interoceptive processing relates to the core features of the condition, including the social affective and restricted and repetitive behavior domains. Here, we used the ADOS-2 (Lord et al. 2012) to examine the relationship between interoceptive dimensions (interoceptive accuracy, interoceptive sensibility, ITPE) and autism severity. Specifically, we hypothesized that calibrated severity scores on the ADOS-2 would be (a) negatively related to interoceptive accuracy scores, such that lower interoceptive accuracy should be seen in those with greater autism severity, and (b) positively related to interoceptive sensibility, such that those with greater autism severity should report the highest beliefs about their interoceptive ability (operationalized by subjective ratings of awareness and confidence on heartbeat detection tasks). In turn (c) these differences should result in a positive relationship between autism severity scores and ITPE, with the greatest mismatch between ability and belief in those with the most severe features. Additionally, based on evidence that social and affective performance is linked to interoception (e.g., Shah et al. 2017), and at present, a lack of evidence of a link between repetitive restricted behaviors and interoception, we hypothesized that interoceptive differences should relate more strongly to the symptom dimension of social affect (SA), than restricted and repetitive behaviors (RRB).

## Method

### Participants

Forty-nine children and adolescents were recruited through community contacts in the South East of England. All of these participants had previously received an independent clinical diagnosis of an autism spectrum condition according to the Diagnostic and Statistical Manual of Mental Disorders (DSM; ​American Psychiatric Association 2000, 2013) or International Statistical Classification of Diseases and Related Health Problems 10th Revision (ICD-10; World Health Organization, WHO 1993). Ethical approval was granted by the local Ethics Review Board and all procedures were conducted in accordance with the Declaration of Helsinki. Parents of all children provided written informed consent for them to take part and the children gave their assent.

Participants ranged in age from 6 to 19 years. Forty participants were male and nine were female, reflecting the gender disparity in the diagnosis of autism (Werling and Geschwind 2013). To assess participants’ current autism severity, all participants completed the Autism Diagnostic Observation Schedule—2nd edition (ADOS-2; Lord et al. 2012). Social Communication Questionnaire (SCQ; Rutter et al. 2003) data were available for 43 participants. All participants apart from two currently met criteria for a diagnosis of an ASC on at least one of these measures.^1^ Participant characteristics are reported in Table 1.

**Table 1:** Participant characteristics

Characteristic	Range	Mean	Standard deviation
Age^a^	6–19	12.796	2.915
IQ^a^	64–132	100.449	16.378
Verbal IQ^a^	62–135	99.265	16.711
Perceptual IQ^a^	52–158	101.857	21.111
SCQ^b^	8–36	21.000	6.956
Autism severity score^c^	2–10	6.469	2.337
Social affect severity score^c^	2–10	6.510	2.171
Repetitive and restricted interest severity score^c^	1–10	6.531	2.416

Age reflects chronological age and is given in years

^a^IQ characteristics were measured using standardized composite scores on the Wechsler Abbreviated Scales of Intelligence—second edition (WASI-II; Wechsler and Hsiao-pin 2011)

^b^SCQ denotes scores on the Social Communication Questionnaire (Rutter et al. 2003); n = 42

^c^Autism severity score refers to calibrated severity score (CSS) on the Autism Diagnostic Observation Schedule—second edition (ADOS-2; Lord et al. 2012), revised algorithm (minimum score = 1, maximum score = 10) (Gotham et al. 2007, 2009). Higher scores reflect greater severity. Similarly, social affect (SA) and repetitive and restricted behavior (RRB) severity scores refer to the calibrated domain scores on the ADOS-2 and use the same 1–10 scale as the CSS (Hus et al. 2012; Hus and Lord 2014)

## Measures

### Interoceptive Accuracy

Interoceptive accuracy was gauged by the participants’ ability to detect their own heartbeats using a heartbeat tracking task (Dale and Anderson 1978; Schandry 1981) and a heartbeat discrimination task (Katkin et al. 1983; Whitehead et al. 1977)—both suitable for measuring interoceptive accuracy in typical and autistic children aged over 6 years of age (Palser et al. 2018a). For the *heartbeat tracking task*, participants’ heartbeats were monitored via a pulse oximeter with the soft sensor mounting attached to their index finger. Participants were required to count their heartbeats during six randomized time windows of varying length (25, 30, 35, 40, 45, and 60 s) and, at the end of each trial, to report the number of heartbeats detected to the experimenter. For the heartbeat tracking task, participants were given the following instructions: “Without putting your hands on your body, can you count each heartbeat you feel in your body from the time you hear “start” to when you hear “stop”. Count in your head and I will ask you afterwards how many you felt. Some people will not feel any heartbeats—that is OK too. Please just tell me ‘none’ when I ask you if that is the case”.

For the *heartbeat discrimination task*, each trial consisted of ten tones presented at 440 Hz and of 100 ms duration, which were triggered by the heartbeat. Under the asynchronous condition, a delay of 300 ms was inserted, adjusting for the average (~ 250 ms) between the R-wave and the arrival of the pressure wave at the finger (Payne et al. 2006). Tones were thus presented at 250 ms or 550 ms after the R-wave, which correspond to maximum and minimum synchronicity judgements respectively (Wiens and Palmer 2001). At the end of each trial, participants signalled to the experimenter whether they believed the tones to be synchronous or asynchronous with their heartbeats. Each participant was provided with the following instructions: “You will hear ten beeps. Can you tell me if you think the beeps are in sync (at the same time as your heartbeats), or out of sync (at a different time to your heartbeats)?”.

### Interoceptive Sensibility

To assess interoceptive sensibility, mean scores on the child-adapted Awareness section of the Body Perception Questionnaire (BPQ; Palser et al. 2018a; Porges 1993) were calculated for each participant. This subscale incorporates 39 bodily sensations (e.g. stomach and gut pains) and participants indicated their awareness of each sensation using a five-point scale ranging from ‘never’ (1) to ‘always’ (5). Example items include (item 5) “My mouth being dry” and (item 17) “A swollen tummy”. This subjective measure of interoceptive sensibility denotes the participant’s belief in his/her own interoceptive aptitude, irrespective of actual (objectively-determined) interoceptive accuracy. We have previously shown that the chid-adapted questionnaire has adequate reliability in typical and autistic children (Palser et al. 2018a), which was replicated here (Cronbach’s alpha = 0.78).

As alternative measure of interoceptive sensibility, confidence judgements of participants’ beliefs about their performance were taken after each trial of the heartbeat tracking and discrimination tasks. Following each trial, participants were asked to score their confidence on a five-point scale, ranging from *‘I don’t know’* (low confidence) to *‘I’m sure’* (high confidence). The scale was illustrated using schematic faces (see Supplementary Fig. 1). Each face corresponded to a score of between 1 (low confidence) and 5 (high confidence).

### Interoceptive Trait Prediction Error

The ITPE was defined operationally as the difference between objective interoceptive accuracy and subjective interoceptive sensibility. For each interoceptive accuracy and sensibility variable (heartbeat tracking score, heartbeat detection score, and Awareness subsection of the BPQ), scores were converted to standardized z-scores. On a within-participant basis, ITPE values were calculated as the difference between interoceptive sensibility and interoceptive accuracy. ITPEs were calculated separately using accuracy scores from each task (heartbeat tracking ITPE_T_ and heartbeat discrimination ITPE_D_), using in each case the sensibility score provided by the BPQ. Positive values of ITPE indicate a propensity for individuals to overestimate their interoceptive ability, while negative scores a propensity to underestimate their own interoceptive ability.

### Autism Diagnostic Observation Schedule

Autism severity was measured in autistic children using the Autism Diagnostic Observation Schedule—second edition (ADOS-2; Lord et al. 2012). The ADOS-2 is a standardized observational scale, administered by a trained examiner, designed to provide opportunities or ‘presses’ for the evaluation of social, communicative and repetitive behaviors, lasting approximately 40 min. In total, 22 participants completed Module 3 of the ADOS-2, and 27 completed Module 4. Calibrated severity scores (Gotham et al. 2007, 2009) and calibrated domain totals for social affect (SA) and restricted and repetitive behaviour (RRB) (Hus et al. 2012; Hus and Lord 2014) were calculated according to published algorithms. Higher scores reflecting greater autism severity (maximum score = 10).

### General Procedure

Following informed consent, all participants completed the BPQ, followed by the heartbeat detection tasks. To prevent the temporal timing of tones priming participants towards their own heart rate, the heartbeat discrimination task was always presented after the heartbeat tracking task. Just prior to starting the heartbeat tracking task, participants were asked to sit quietly and told to focus internally, to try to feel their heart beating. This was repeated a total of six times using a variety of randomized trial lengths (25, 30, 35, 40, 45, and 60 s). Once this task was completed, participants then performed the heartbeat discrimination task. This procedure was repeated ten times. Assessments of intellectual functioning, using the Wechsler Abbreviated Scales of Intelligence—second edition (WASI-II; Wechsler and Hsiao-pin 2011), and autism severity, using the ADOS-2, were administered to all children.

### Statistical Analysis

To derive measures for interoceptive accuracy, heartbeat tracking scores were calculated on a trial-by-trial basis according to the ratio of perceived to actual heartbeats: 1 − |nBeatsReal − nBeatsReported|/(nBeatsReal + nBeatsReported)/2 (Garfinkel et al. 2015; Hart et al. 2013) and these were averaged to form a mean heartbeat tracking score. This measure calculates interoceptive accuracy independent of the number of heartbeats in the trial by normalizing the absolute error in perceived heartbeats as a function of the overall number of heartbeats. Interoceptive accuracy for the heartbeat discrimination task was assessed as a ratio of correct to incorrect synchronicity judgements. Interoceptive sensibility was calculated as mean score on the BPQ and mean confidence ratings on the heartbeat tracking and discrimination tasks.

Multiple hierarchical linear regressions were used to determine the effect of interoceptive dimensions (interoceptive accuracy, sensibility and ITPE) on symptom severity after accounting for participant characteristics (age, sex, IQ). Participant characteristics were entered in block one, and interoceptive dimensions in block two. Standardized IQ t-scores were used in the analysis as no multicollinearity was observed with age. BPQ scores and IPTE_D_ however were excluded from models due to multicollinearity (tolerance < 0.2). The independent contribution of interoception to domain scores, beyond significant demographic predictors, was explored using mixed effects models employing fixed and random effects. Goodness of model fit was quantified using Akaike information criterion (AIC) and models were compared using likelihood ratio tests via analysis of variance (ANOVA).

## Results

### Initial Data Screening

Five participants were missing heartbeat discrimination scores due to equipment failure, therefore estimation of the effects of variables pertaining to this measure involved only 42 participants. Two different participants were unable to complete all trials of the heartbeat tracking task, either due to fatigue or time restrictions, therefore estimation of the effects of variables pertaining to this measure involved only 47 participants. Pairwise deletion was used in analyses to maximize the number of observations available.

### Overall Symptom Severity

**Fig. 1:**
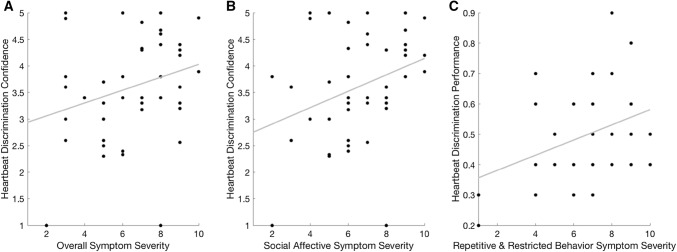
Overall autism symptom severity was positively associated to heartbeat discrimination confidence, tapping interoceptive sensibility (Plot** a**). When symptom domains were considered separately, this relationship was replicated in the social affective domain (Plot** b**). Symptoms in the repetitive and restricted behavior domain, however, were related to heartbeat discrimination performance, tapping interoceptive accuracy (Plot** c**)

The regression model was significant [*F*(8,40) = 3.927, *p *= 0.003]. The most significant predictor of overall symptom severity was sex (*p *= 0.002), followed by heartbeat discrimination confidence (*p *= 0.011) and heartbeat tracking confidence (*p *= 0.049) (see Table 2). Those with the most severe features reported the highest confidence on the heartbeat discrimination task (see Fig. 1a) but also, paradoxically, the lowest confidence on the heartbeat tracking task. Male participants (median = 7) had significantly greater autism severity than female participants (median = 3) on the ADOS-2 [*U *= 50.500, *p *< 0.001] (see also Lai et al. 2011).

**Table 2:** Linear regression analysis indicates that in addition to sex, interoceptive sensibility, as measured with trial-wise confidence judgements on the heartbeat discrimination and tracking tasks, makes an independent contribution to overall autism symptom severity

Predictor	*Β*	*t*	*p*
Sex*	0.445	3.450	0.002
Heartbeat discrimination confidence*	0.449	2.685	0.011
Heartbeat tracking confidence*	− 0.405	− 2.046	0.049
IQ	− 0.286	− 1.953	0.060
ITPE_T_	0.125	0.637	0.529
Heartbeat discrimination	0.071	0.536	0.596
Heartbeat tracking	− 0.062	− 0.273	0.786
Age	0.019	0.129	0.989

Hierarchical linear regression model with the background characteristics of age, sex and IQ entered in block one and interoceptive dimensions of heartbeat tracking performance, heartbeat tracking confidence, heartbeat discrimination performance, heartbeat discrimination confidence, and interoceptive trait prediction error for the heartbeat tracking task (ITPE_T_) entered in block two. Standardized beta coefficients are reported. Asterisks indicate significant predictors at the *p * < 0.05 alpha level

The model accounted for 36.9% of the variance in overall symptom severity (Adjusted R^2^ = 0.369). Sex explained 24.1% of the variance in symptom severity, and interoceptive sensibility (confidence judgements on heartbeat discrimination and tracking tasks) explained an additional 13.1%.

### Social Affective Domain

The regression model was significant [*F*(8,40) = 3.790, *p *= 0.003] and indicated that the most significant predictor of social affective (SA) symptoms was again sex (*p *= 0.001), followed by heartbeat discrimination confidence (*p *= 0.011) (see Table 3). Greater severity in the SA domain was associated with higher confidence on the heartbeat discrimination task (see Fig. 1b). This model accounted for 35.8% of the variance in SA symptoms (Adjusted R^2^ = 0.358). Alone, sex explained 23.9% of the variance in SA symptoms and interoceptive sensibility explained 8.3%.

**Table 3:** Linear regression analysis indicates that in addition to sex, interoceptive sensibility, as measured with trial-wise confidence judgements on the heartbeat discrimination task, makes an independent contribution to autism symptom severity in the social affective domain

Predictor	*Β*	*t*	*p*
Sex*	0.456	3.502	0.001
Heartbeat discrimination confidence*	0.458	2.718	0.011
Heartbeat tracking confidence	− 0.306	− 1.532	0.135
IQ	− 0.216	− 1.462	0.153
ITPE_T_	0.193	0.978	0.335
Heartbeat tracking	− 0.177	− 0.772	0.446
Age	0.042	0.287	0.776
Heartbeat discrimination	0.005	0.034	0.973

Hierarchical linear regression model with the background characteristics of age, sex and IQ entered in block one and interoceptive dimensions of heartbeat tracking performance, heartbeat discrimination performance, heartbeat tracking confidence, heartbeat discrimination confidence, and interoceptive trait prediction error for the heartbeat tracking task (ITPE_T_) entered at the second level. Standardized beta coefficients are reported. Asterisks indicate significant predictors at *p * < 0.05.

Due to the large effect of sex on SA symptoms, the data were explored further using mixed effects regression models. In line with the results of the hierarchical linear regression, including a random intercept for each sex significantly improved model fit (AIC = 187.03) compared to a baseline model only including the intercept and error term (AIC = 192.6) (likelihood ratio = 7.472, *p *= 0.006). As predicted, adding a fixed effect of heartbeat discrimination confidence significantly improved the model further (AIC = 184.219) (likelihood ratio = 4.819, *p *= 0.028).

### Repetitive and Restricted Behavior Domain

The regression model did not meet the threshold for significance [*F*(8,40) = 2.078, *p *= 0.068]. The only significant predictor of severity in the repetitive and restricted behavior (RRB) domain was heartbeat discrimination performance (*p *= 0.013). More accurate interoception was seen in those with greater autism severity (Table 4).

**Table 4:** Linear regression analysis indicated that the only significant predictor of symptom severity in the repetitive and restricted behavior domain was heartbeat discrimination performance

Predictor	*Β*	*t*	*p*
Heartbeat discrimination*	0.399	2.630	0.013
ITPE_T_	0.307	1.372	0.180
Sex	0.188	1.274	0.212
Age	0.204	1.229	0.228
Heartbeat discrimination confidence	− 0.202	− 1.058	0.298
IQ	− 0.128	− 0.766	0.449
Heartbeat tracking	− 0.128	− 0.494	0.625
Heartbeat tracking confidence	− 0.095	− 0.419	0.678

Hierarchical linear regression model with the background characteristics of age, sex and IQ entered in block one and interoceptive dimensions of heartbeat tracking performance, heartbeat discrimination performance, heartbeat tracking confidence, heartbeat discrimination confidence, and interoceptive trait prediction error for the heartbeat tracking task (ITPE_T_) entered at the second level. Standardized beta coefficients are reported. Asterisks indicate significant predictors at *p * < 0.05

To summarize, overall autism severity, as measured with the ADOS-2, was significantly positively related to interoceptive sensibility, as measured by confidence judgements on the heartbeat discrimination task. This relationship was predominantly driven by severity of features in the social-affective domain. Severity of repetitive restricted behaviors was only significantly positively related to interoceptive accuracy on the heartbeat tracking task. Surprisingly, there was also a weakly significant negative relationship between overall autism severity and interoceptive sensibility as measured by confidence judgements on the heartbeat tracking task.

## Discussion

In this study, we hypothesized that there would be a relationship between core autism features and interoceptive differences in autism. Specifically, we predicted that we would observe decreasing interoceptive accuracy but increasing interoceptive sensibility as autism severity increased. This would result in a positive association between interoceptive trait prediction error (ITPE)—the discrepancy between interoceptive accuracy and interoceptive sensibility—and core autistic features, especially in the social affective (SA) domain.

In partial support of our hypotheses, the results indicated that there was a positive association between autism severity, as measured using calibrated severity scores from the ADOS-2 (Lord et al. 2012) and interoceptive differences in the domain of interoceptive sensibility. Higher judgements of confidence on the heartbeat discrimination task were linked to greater autism severity. As predicted, this finding was driven by features in the SA domain; when domains were separated, the association was replicated in this but not the RRB domain. Counter to our hypotheses, interoceptive accuracy on the heartbeat discrimination task related positively to symptoms in the RRB domain. Furthermore, and also inconsistent with our hypotheses, there was a weakly significant negative correlation between confidence on the heartbeat tracking task and overall autism severity. These findings suggest that the different autism feature clusters might diverge in their relationships to distinct aspects of interoceptive processing.

Inflated confidence in interoceptive judgements on the heartbeat discrimination task was associated with greater SA severity. Heightened interoceptive sensibility has previously been linked to alexithymia, a difficulty with reasoning about one’s own affective state (Mul et al. 2018), and anxiety (Palser et al. 2018b). Previously, a combination of high confidence in interoceptive ability and poor performance has been observed in autistic participants (Garfinkel et al. 2016; Palser et al. 2018a). In addition, we also observed evidence that low confidence in interoceptive judgements on a different task was associated with greater symptom severity. Taken together, these findings suggest that subjective confidence might represent a noisier channel of information, at least in children on the autism spectrum, with a difficulty mapping belief to performance.

Autism has previously been framed as a ‘disorder of metacognition’, resulting from altered beliefs about the reliability of sensory inferences (Lawson et al. 2014). This might reflect a reduced influence of prior experience on current estimations (Pellicano and Burr 2012) or inflexible predictions about current states (Van de Cruys et al. 2013). Reduced insight into one’s access to interoceptive states may be linked to a reduced ability to infer these states in others. It has previously been suggested that similar systems may support both the ability to self-reflect and infer the thoughts, beliefs and behaviors of others (Carruthers 2009, Frith and Frith 2001; Frith and Happé 1999; Mitchell et al. 2005). As such, good metacognitive insight, including interoceptive insight, may scaffold the development of social competencies, particularly the ability to mentalize about others. The finding of an association between interoceptive confidence judgements and autism features, in combination with the lack of association to interoceptive accuracy, which is thought to measure an individual’s (conscious) access to these signals, suggests it may be the confidence placed in the detection of that interoceptive signal and not the detection of the interoceptive signal itself that is of most importance to many of the core features of autism.

Unfortunately, we were not able to calculate scores for interoceptive awareness, the third dimension in Garfinkel and colleagues’ interoception model. Such analyses generally require the calculation of the area under the receiver operating characteristic (ROC) curve, which quantifies in one value, how well confidence follows accuracy. These methods originated in the exteroceptive perceptual metacognition literature where they were most often applied to visual perception tasks (e.g. Fleming et al. 2010). Extending the confidence scale to sufficient extent that robust calculation of ROC curves would have been possible was judged to be too complicated for younger participants. Only one study to date has measured interoceptive awareness in autistic participants, and found no significant differences in adults diagnosed with the condition, relative to neurotypical adults (Garfinkel et al. 2016). Nonetheless, further work should seek to examine if individual differences in this interoceptive dimension relate specifically to social and affective understanding.

The positive relationship between interoceptive accuracy and RRB symptoms is curious, and, at first glance, counter to previous findings of reduced interoceptive accuracy in autism (Garfinkel et al. 2016; Mul et al. 2018; Nicholson et al. 2019; Palser et al. 2018a). Both high performance on the heartbeat tracking task and engagement in RRBs require highly focused attention while ignoring external distractors. It is possible that the children who are most attentive in this manner, due to elevated RRBs, also represent those most capable of focussing their attention on cardiac signals when instructed. RRBs are often thought of as maladaptive, distracting the child from social engagement or learning. However, there is evidence to suggest that some of the RRBs in which autistic children frequently engage, such as hand flapping and body rocking, are self-soothing and may have an adaptive function as a means of lowering arousal and regulating the autonomic nervous system (which generates interoceptive signals) (Hutt et al. 1975; Kapp et al. 2019).

RRB features were only predicted by interoceptive accuracy on the heartbeat discrimination task, not the heartbeat tracking task. Previously, the latter has been the most sensitive measure of interoceptive accuracy differences in autistic people (Garfinkel et al. 2016; Mul et al. 2018; Palser et al. 2018a). However, there has recently been increasing criticism of the heartbeat tracking task due to the necessity of multiple control measures and its relatively poor psychophysical properties (Murphy et al. 2018; Zamariola et al. 2018). These concerns may explain the variability in this measure across studies. There are also limitations associated with the heartbeat discrimination task. This task may not be a pure interoceptive measure, as it requires the temporal integration of an interoceptive signal (the heartbeat) with an exteroceptive signal (the tone). Differences in multisensory integration have been well-documented in autism (e.g., Brandwein et al. 2015) and, as such, it is interesting that no group differences have yet been observed on this measure.

Much like the broader literature on interoception, studies of interoceptive processing in autism have almost exclusively focussed on cardiac interoception. Many of the reasons for this focus are pragmatic. The heartbeat represents a discrete regular event that is easily and non-invasively recorded. Most individuals experience at least some conscious awareness of the signal and can easily compare detected versus occurred events. Aside from these practical issues, the popularity of heartbeat detection paradigms rests in part on the assumption that interoceptive accuracy represents an overall or general interoceptive capacity. This assumption may mask important differences in autism between sources of interoceptive information. While in the cardiac domain, autistic individuals are more likely to experience reduced awareness, an aversion to social touch is frequently seen in infants who later obtain an autism diagnosis, suggesting heightened perception in this domain (Baranek 1999; Grandin and Scariano 1986). Interoceptive functioning should now be assessed in autism using other measures, such as tests of water load and taste sensitivity (Murphy et al. 2018; Van Dyck et al. 2016) to assess if the present findings of an association between cardiac interoception and core autism features generalize to other interoceptive systems.

Some authors argue that any interoceptive differences seen in autism are in fact the result of high levels of comorbid alexithymia, rather than autism itself (Gaigg et al. 2018; Shah et al. 2016). Studies indicate that around 50% of autistic people also show clinically meaningful levels of alexithymia (Griffin et al. 2016; Hill et al. 2004). As alexithymia involves the individual’s experience of their own emotions, in the vast majority of cases it is measured using a self-report questionnaire. Accurate completion of such a measure therefore requires sufficient insight on the part of the participant. That is, they must be aware that they are experiencing difficulties with their emotions, and that other people’s experiences are different, in order to report such difficulties. The validity of such measures in children, particularly autistic children, is unclear. The only study to have used an alexithymia measure with children yielded no significant correlation between parent- and child-rated alexithymia in this population (Griffin et al. 2016). For this reason, a measure of alexithymia was not included in the current study. That being said, some of the emotional presses in the ADOS-2 appear to tap alexithymic difficulties, by testing how well the participant describes the experience of several emotions. Some recent investigations have failed to show a relationship between alexithymia and interoceptive differences in autistic people (Nicholson et al. 2018, 2019) and the present study demonstrates a link between the non-emotional features of autism (RRBs) and interoception, suggesting alexithymia may not explain the entirety of interoceptive differences in autism. Nevertheless, the absence of measures of alexithymia and anxiety in the current study, which have been previously found to be associated with interoception in autism, are potential limitations of the current study. Future work should seek to explore this question further, with the development of validated measures of alexithymia in autistic child populations.

Some further limitations of the study are discussed below. Interoception only explained a small amount of the variance in symptom severity, Indeed, the sex of participants explained more variance at both the level of overall symptoms and in the SA domain. Each symptom domain reflects a broad range of behaviors, and is likely shaped by multiple biological, psychological and situational factors. Given such a diverse set of antecedents, any one factor, at any particular level of analysis, will likely explain only a fraction of an individual’s autistic features. The present findings suggest that interoceptive processing may represent one psychological antecedent, although further work is now needed to establish a causal link. Indeed, theoretical accounts that posit a role for atypical interoception in autism are causal models, explicitly relating interoception to the development of autism (Quattrocki and Friston 2014). The correlational data presented here, while consistent with the predictions of these hypotheses, do not offer sufficient evidence to support such a causal claim. Longitudinal research beginning early on in development, and the manipulation of interoceptive processing while observing the effect on autistic features, will be necessary to further our understanding of the relationship between autism and interoception.

Taken together, the current findings extend existing findings showing a link between interoception and the co-occurring features of autism (namely anxiety), to the core features of the condition, in both the domains of social and affective processing and repetitive and restricted behaviors. However, these domains relate to distinct aspects of interoceptive processing suggesting that considering autism severity as a single dimension may mask important relationships between autistic features and interoception. If replicated, the divergence between how distinct symptom clusters relate to different domains of interoceptive processing suggests that we may need to tailor interventions to individual interoception-symptom profiles.

## Electronic supplementary material

Below is the link to the electronic supplementary material.

Supplementary material 1 (DOCX 191 kb)

## Footnotes

1 Excluding these two participants did not alter the relationships observed between autism symptom dimensions and interoceptive variables and so they were retained in the sample for all subsequent analyses.
