# Supplementary material for: Dissociation in How Core Autism Features Relate to Interoceptive Dimensions: Evidence from Cardiac Awareness in Children
Source: J Autism Dev Disord. 2019 Nov 9;50(2):572–82. doi: 10.1007/s10803-019-04279-4 (PMC6995984; doi:10.1007/s10803-019-04279-4)

**Supplementary Materials**

Supplementary Figure 1: Participants indicated their trial-wise confidence on the heartbeat tracking and discrimination tasks using a five-point scale illustrated using schematic faces to denote total certainty (“I’m sure) (5) to minimal certainty (“I don’t know”) (1).


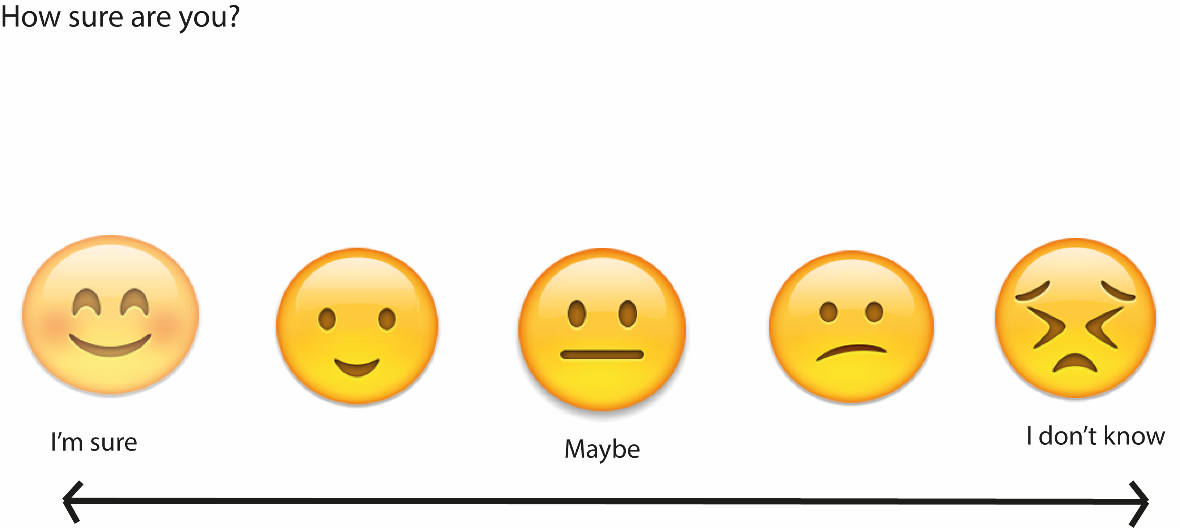

Supplement: Supplementary file 1 — Supplementary material 1 (DOCX 191 kb) [file 10803_2019_4279_MOESM1_ESM.docx]
